# Power of Combined Intravenous and Intraventricular Colistin Against Multidrug-Resistant Bacteria in Healthcare-Associated Ventriculitis and Meningitis

**DOI:** 10.7759/cureus.96359

**Published:** 2025-11-08

**Authors:** Sanae Azelmat, Tarik Baadi, Hicham Hammadi, Nawfal Doghmi, Mariama Chadli

**Affiliations:** 1 Bacteriology, Mohammed V Military Training Hospital, Rabat, MAR; 2 Anesthesiology and Critical Care, Mohammed V Military Training Hospital, Rabat, MAR

**Keywords:** colistin, healthcare-associated ventriculitis and meningitis, intravenous, intraventricular, klebsiella pneumoniae, multidrug-resistant

## Abstract

Healthcare-associated ventriculitis and meningitis (HAVM) caused by multidrug-resistant (MDR) gram-negative organisms represents a major therapeutic challenge, given the limited penetration of most antibiotics into the cerebrospinal fluid (CSF). Colistin, although active against MDR strains, exhibits poor CSF distribution when administered intravenously alone.

We report a case of a 74-year-old man admitted with a cerebellar hemorrhage complicated by hydrocephalus requiring external ventricular drainage. The patient subsequently developed HAVM due to MDR *Klebsiella pneumoniae*. Initial systemic antibiotic regimens, including intravenous (IV) colistin, failed to control the infection. A combined strategy with IV and intraventricular (IVT) colistin was initiated, resulting in rapid improvement of inflammatory biomarkers and microbiological clearance. However, the patient ultimately died from neurological complications related to the primary hemorrhagic stroke.

This case illustrates the potential benefit of combined IV and IVT colistin administration in refractory MDR gram-negative HAVM. While clinical outcomes may be limited by underlying neurological severity, adjunct IVT therapy ensures effective CSF antibiotic concentrations and may improve infection control. Further research is needed to optimize dosing and establish standardized treatment protocols.

## Introduction

Healthcare-associated ventriculitis and meningitis (HAVM) is a devastating infection that occurs after neurosurgical interventions, including external ventricular drain (EVD) placement. Gram-negative bacilli, notably *Klebsiella pneumoniae*, are the most frequent causative organisms [1-3]. Multidrug-resistant (MDR) pathogens are defined as resistant to at least three antimicrobial classes, whereas extensively drug-resistant (XDR) strains remain susceptible to only one or two classes of antibiotics [4]. The isolation of MDR organisms in postoperative central nervous system (CNS) infections has increased globally, with *K. pneumoniae* being reported more frequently in ventriculitis and meningitis cases [5,6]. Treatment of HAVM remains challenging because most antibiotics have limited penetration across the blood-brain barrier. Colistin, a polymyxin antibiotic, demonstrates poor CSF diffusion when administered intravenously, with reported CSF/serum ratios as low as 0.07 [7]. Therefore, adjunctive intraventricular (IVT) administration may achieve therapeutic CSF concentrations, improving outcomes in MDR infections [8]. Mortality in HAVM remains high, ranging from 15% to 70%, underscoring the urgency for early and effective therapy [9]. Here, we report a case of HAVM caused by MDR *K. pneumoniae *that was successfully treated with a combination of intravenous (IV) and IVT colistin after IV therapy alone failed, highlighting the importance of tailored antimicrobial strategies in critically ill patients.

## Case presentation

A 74-year-old man with a poorly controlled history of arterial hypertension presented to the emergency department after a fall from standing height, exhibiting paraparesis and dysarthria. Initially, he had weakness in both lower limbs and mild upper limb deficits. Within hours, his neurological status deteriorated to paraplegia with significant upper limb weakness.

**Figure 1:**
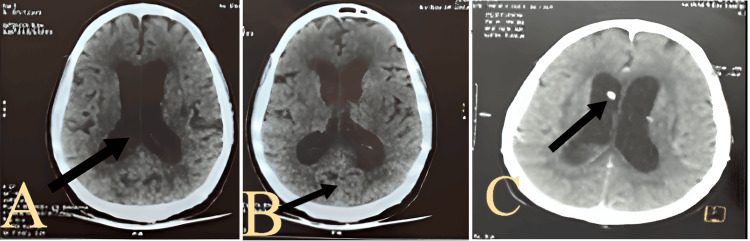
Axial non-contrast cranial CT scan showing moderate triventricular hydrocephalus (A, arrow), left cerebellar and vermian hemorrhagic stroke (B, arrow), and a drain in place (C, arrow). These images support the diagnosis of the patient described in the case presentation. The patient subsequently required placement of an EVD for CSF diversion. EVD: external ventricular drain; CT: computed tomography; CSF: cerebrospinal fluid

A cranial non-contrast computed tomography (CT) scan revealed a left cerebellar and vermian hemorrhagic stroke with moderate triventricular hydrocephalus (Figure 1). The patient was intubated and mechanically ventilated for airway protection and transferred to the intensive care unit (ICU). An EVD (Spiegelberg GmbH & Co. KG, Germany) was placed for CSF diversion.

Empiric therapy with amoxicillin-clavulanic acid was initiated for suspected aspiration pneumonia. On day six post-admission, he developed a fever of 38.8°C and elevated inflammatory markers (C-reactive protein (CRP) 156 mg/L). CSF analysis showed protein 1.8 g/L, glucose 0.8 mmol/L (serum glucose 5.2 mmol/L), and 620 cells/mm³ with 90% neutrophils, consistent with bacterial meningitis (Table 1). Vancomycin and meropenem were started empirically.

**Table 1:** Laboratory and CSF findings on the day of diagnosis. Laboratory and CSF parameters demonstrate neutrophilic pleocytosis, hypoglycorrhachia, and hyperproteinorrachia. CSF: cerebrospinal fluid; CRP: C-reactive protein

Parameter	Patient Value	Units
Temperature	38.8	°C
White blood cell count	14.2	×10⁹/L
Hemoglobin	11.5	g/dL
Platelet count	270	×10⁹/L
CRP	156	mg/L
Serum glucose	5.2	mmol/L
CSF protein	1.8	g/L
CSF glucose	0.8	mmol/L
CSF/serum glucose ratio	0.15	-
CSF white cell count	620 (90% neutrophils)	cells/mm³

Within 24 hours, CSF culture identified MDR *K. pneumoniae*, resistant to cephalosporins, carbapenems, and aminoglycosides, but susceptible to colistin. Therapy was switched to IV colistin (loading dose 9 million IU, maintenance 4.5 million IU every 12 hours) combined with daily IVT colistin (125,000 IU).

During treatment, inflammatory biomarkers decreased, and repeat CSF analyses demonstrated a reduction in cell count and protein levels. Clinically, the patient remained neurologically impaired with minimal improvement in motor function. No major nephrotoxicity or neurotoxicity related to colistin was observed. Despite microbiological clearance, the patient eventually succumbed to complications of the initial hemorrhagic stroke.

## Discussion

Healthcare-associated ventriculitis and meningitis represent severe complications after neurosurgical interventions, with the incidence of EVD-related infections reported between 0% and 22% [2]. MDR and XDR organisms are increasingly observed, complicating management due to limited antimicrobial options and poor blood-brain barrier penetration [3,4].

*K. pneumoniae*, a gram-negative bacillus and gut commensal, can cause life-threatening CNS infections when resistant strains are involved [5,6]. Reports indicate rising MDR strains resistant to cephalosporins, aminoglycosides, and carbapenems, leaving colistin as one of the few effective options [7-9].

Colistin’s poor CSF penetration via IV administration necessitates adjunctive IVT therapy. Studies have demonstrated that combined IV and IVT colistin improves pharmacokinetic efficacy in the ventricular and subarachnoid spaces, often leading to more rapid microbiological clearance [8,10]. In our patient, IVT administration achieved clinical and laboratory improvement, confirming the potential benefit of direct CSF delivery in MDR infections.

Despite aggressive therapy, outcomes may remain poor due to the severity of the primary neurological damage, as seen in this case. Mortality in HAVM varies widely, from 15% to 70%, depending on the pathogen, patient comorbidities, and timeliness of appropriate therapy [9,11]. This underscores the need for early detection, microbiological confirmation, and tailored antimicrobial regimens.

Future research should aim to standardize IVT colistin dosing, evaluate combination therapies, and explore prophylactic strategies for high-risk neurosurgical patients to reduce the incidence of MDR CNS infections [12,13].

## Conclusions

This case highlights the therapeutic potential of combined IV and IVT colistin in treating HAVM caused by MDR *K. pneumoniae*. Despite an unfavorable neurological outcome, the rapid biochemical response underscores the role of IVT therapy in achieving effective CSF concentrations when systemic therapy fails. Further research is warranted to optimize dosing and establish standardized protocols.
